# Observation of the Specific Heat Jump in the Se-Substituted MoTe_2_ Single Crystals

**DOI:** 10.3390/ma15113782

**Published:** 2022-05-25

**Authors:** Aoi Kobayashi, Yoshiki Takano, Satoshi Demura

**Affiliations:** College of Science and Technology, Nihon University, Chiyoda-ku, Tokyo 101-8308, Japan; csao20009@g.nihon-u.ac.jp (A.K.); takano.yoshiki@nihon-u.ac.jp (Y.T.)

**Keywords:** superconductivity, transition metal chalcogenides, transport properties

## Abstract

1T’-MoTe_2_ has gained considerable attention owing to its topological character. This material undergoes spatial inversion symmetry at 300 K. A structural transition to the T_d_ phase, which is represented by a kink in the resistivity, was observed below 250 K without inversion symmetry along the *c*-axis, while superconductivity was observed at 0.1 K. Substitution of Se into this material suppressed the appearance of the kink structure and increased the superconducting transition temperature to 2 K, which is consistent with previously reported results on polycrystalline samples. However, a specific heat jump was observed in the obtained single crystals, which did not exhibit kink structures in their resistivity. The results suggest that the T_d_ structure was not suppressed entirely after Se substitution and that superconductivity was achieved without inversion symmetry.

## 1. Introduction

Transition metal chalcogenides have been extensively studied owing to their unique properties. The properties of such compounds depend on crystal polymorphism. MoTe_2_, which is a chalcogenide, may undergo different crystal polymorphisms: 2 H, 1T’, and T_d_ types [1,2,3,4]. The 2 H type represents a semiconducting electronic structure, whereas the 1T’ type implies a semimetallic state. The 1T’ type changes to the T_d_ type at temperatures below 250 K. The crystal structure of the T_d_ type does not have spatial inversion symmetry along the *c*-axis [1]. Owing to symmetry breaking, the T_d_ type has been predicted to be type-II Weyl semimetals [5]. Furthermore, the T_d_ type exhibits superconductivity at 0.1 K [6]. Therefore, 1T’- and T_d_- type MoTe_2_ are useful for topological physics research to determine the relationship between the topological character and superconductivity [7]. Superconductivity is enhanced via the application of pressure and element substitution [6,8,9,10,11]. However, avoiding a transition to the T_d_ type is also necessary. Because T_d_-type suppression results in the recovery of inversion symmetry, the relationship between the enhancement and the topological state remain unclear. To resolve this issue, it is useful to confirm whether the structural phase-transition is suppressed in the bulk. 

In this study, we performed specific heat measurements in Se-substituted MoTe_2-*x*_Se*_x_* single crystals. The Se concentration was finely tuned in these crystals, which were prepared using the chemical vapor transport method. The kink in the resistivity curve related to the structural transition was observed at approximately 290 K for *x* = 0.0–0.12, and it was suppressed for *x* > 0.12. The onset of the superconducting transition temperature increased to approximately 3 K for *x* = 0.13–0.28. These results are similar to those previously reported for polycrystalline Se-substituted MoTe_2_ [10]. Moreover, a specific heat jump for all samples was observed at approximately 290 K. This observation is similar to the observed kink for the *x* = 0.0–0.07 samples. These results indicate that the structural transition is not entirely suppressed. Therefore, the superconductivity is enhanced by maintaining the T_d_-type structure possibility.

## 2. Experimental Details

**Figure 1:**
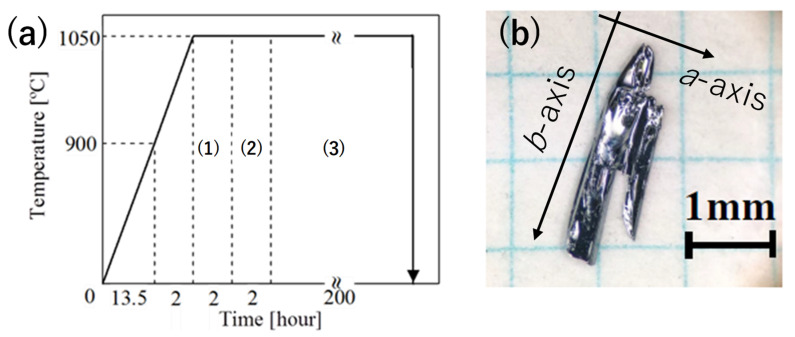
(**a**) Thermal conditions to obtain 1T’-MoTe_2_. (1–3) indicate the stabilizing furnace, preparation slope, and temperature stability processes, respectively. At first, the furnace was heated up to 1050 °C for 15.5 h and maintained for 2 h for temperature stabilization (1). Next, the high and low-temperature ends were set to 1050 °C and 950 °C for 2 h, respectively (2). The temperature condition was maintained for 200 h (3). Thereafter, quartz tubes were immersed in water to avoid 2 H structure formation (hexagonal crystal), which is known to be stable at approximately 800 °C. (**b**) Image of the 1T’-MoTe_2_ single crystal sample prepared by chemical vapor deposition.

Single crystals of MoTe_2-*x*_Se*_x_* were prepared by chemical vapor transport. Mo (purity 99.9%), Te (purity 99.9%), and Se (99.9%) powders were used as raw materials. The aforementioned powders was used following the stoichiometric ratio of MoTe_2-*x*_Se*_x_*. The weight of these powders is 1 g. I_2_ (5 g/ml) was used as the transport material. Owing to the volatility of I_2_, it was weighed immediately before proceeding to the evacuation process. The raw materials and I_2_ were sealed in an evacuated quartz tube (4.0 × 10^−3^ Pa). A three-zone horizontal electric furnace (ARF3-600, Asahi-rika Co., Chiba, Japan) was used to sinter the evacuated quartz tube. The thermal preparation process is illustrated in Figure 1a. The dimensions of the prepared samples were 2 × 3–5 mm^2^, as depicted in Figure 1b. These crystals contained metallic silver, similar to the 1T’-MoTe_2_ crystals previously reported. Additionally, these crystals easily glowed along the *b*-axis [4]. Therefore, the longitudinal direction corresponded to the *b*-axis, as shown in Figure 1b.

X-ray diffraction (XRD, Ultima IV, Rigaku, Tokyo, Japan) measurements used by a Cu-Kα radiation (λ = 1.54056 Å) were performed for single and powdered samples, wherein the single crystals were ground. Simulation peaks were calculated using VESTA (version 3.4.4, Japan) [12]. Crystal structure data for 1T’-MoTe_2_ were obtained from AtomWorks (http://crystdb.nims.go.jp/, accessed on 8 August 2019) [13]. Electron scanning microscopy (SEM, Hitachi High-Tech Fielding Corporation, Tokyo, Japan) and energy dispersive X-ray analysis (EDX, HORIBA, Tokyo, Japan) were conducted to investigate the compositions of the samples. The composition ratios of Mo, Te, and Se were determined, and the composition of Mo was normalized to 1. Electrical resistivity and specific heat were measured using a Physical Property Measurement System (PPMS, Quantum Design, Takamatsu, Japan). Because the resistivity values for the MoTe_2_ samples are relatively low, a four-terminal method was adopted. The temperature range was set to 2–300 K. A heat relaxation method was used for specific heat measurements in the range of 3–300 K.

## 3. Result and Discussion

**Figure 2:**
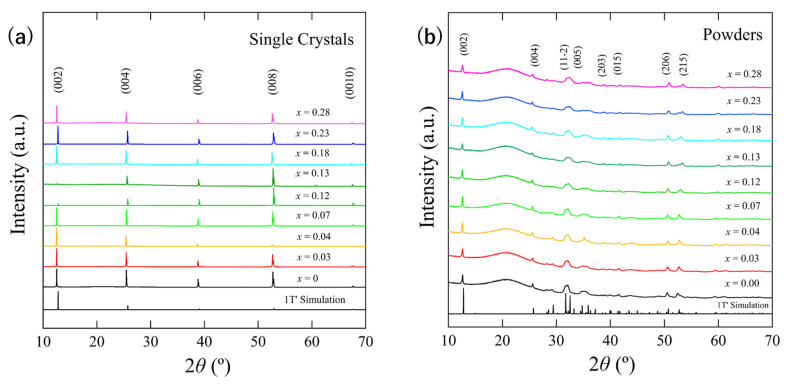
Results of XRD measurements of MoTe_2-*x*_Se_x_ samples with (**a**) single crystals and (**b**) powders. Simulation peaks were calculated using VESTA (version 3.4.4, Japan) [12] and crystal data were obtained from AtomWorks (http://crystdb.nims.go.jp/, accessed on 8 August 2019) [13]. The Se concentration (*x*) was obtained by EDX.

XRD measurements were performed on MoTe_2-*x*_Se*_x_* single crystals and powders. The results for both set of samples are presented in Figure 2. The observed peaks were indexed via comparison with the 1T’-type structure simulation. Consequently, the results confirmed that 1T’-MoTe_2-*x*_Se*_x_* single crystals were successfully obtained.

**Figure 3:**
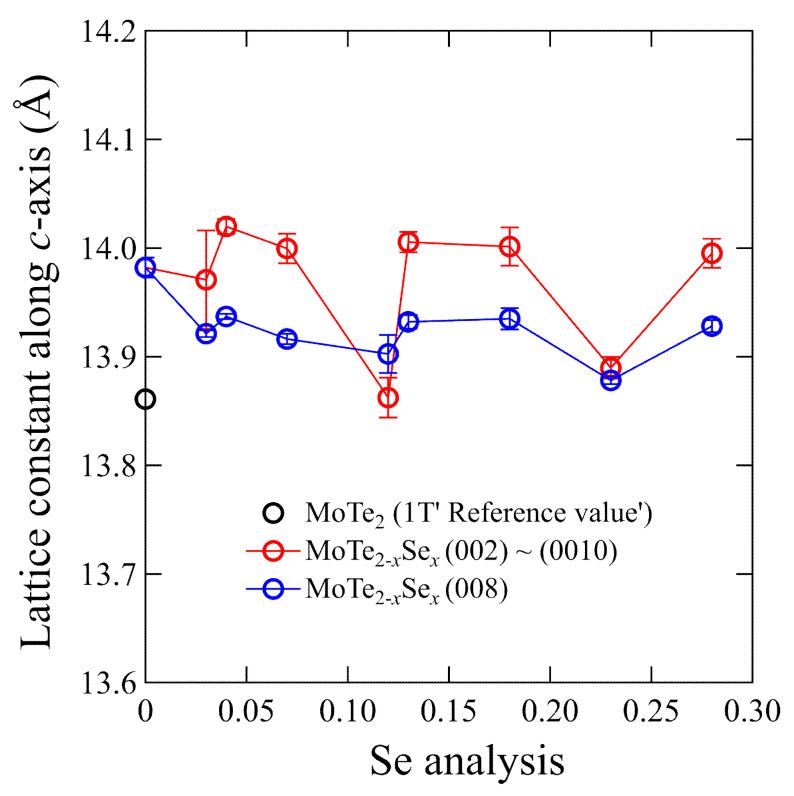
Lattice constants along the *c*-axis calculated through XRD patterns of the (00*l*) plane for single crystals. Error bars were calculated using five samples from the same batch. The reference value curve corresponds to previously reported results [14].

The dependence of the lattice constant on the Se concentration (*x*) along the *c*-axis is depicted in Figure 3. Lattice constants were determined for the (00*l*) (red open circles) and (008) (blue open circles) peaks. The (008) peaks contribute less to the error resulting from the difference in the sample shape, such as the height or roughness of the surface. When Se ions were substituted, the lattice constant along the *c*-axis expects to decrease because the ionic radius of Se is smaller than that of Te. However, the lattice constant was almost constant for all the samples; hence, the lattice constant was not Se-concentration dependent. These results are similar to those reported for S-substituted single crystals and Se-substituted polycrystals [8,10].

**Figure 4:**
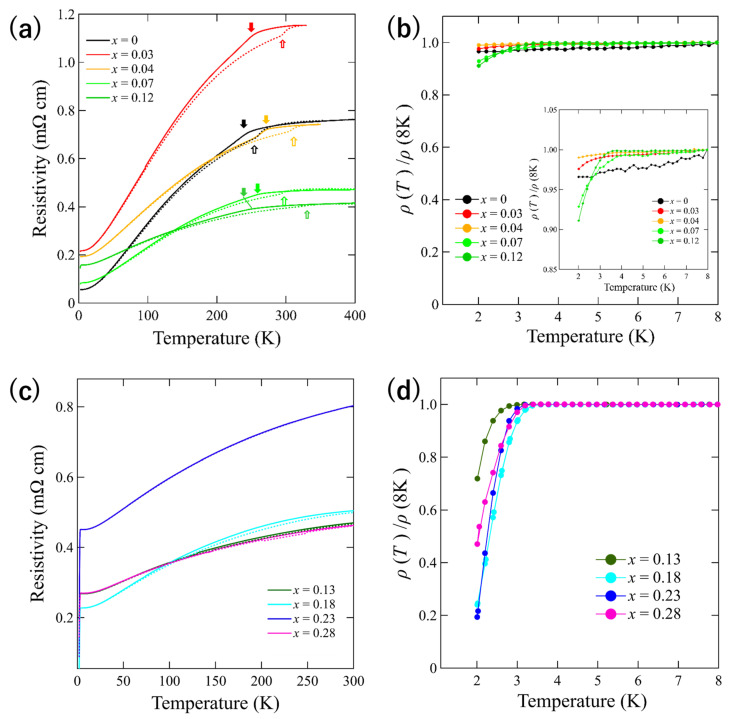
Temperature dependence of electrical resistivity for MoTe_2-*x*_Se*_x_* single crystals. The results for *x* = 0–0.12 and *x* = 0.13–0.28 have been reported. (**a**,**c**) depict results in the entire temperature range. The solid and dotted lines correspond to the cooling and heating processes, respectively. Arrows indicate the temperature at which the kink appears (*T*_s_). Filled and open arrows in (**a**) indicate *T*_s_ for the heating and cooling processes, respectively. (**b**,**d**) illustrate the region below 8 K, normalized by the resistivity at 8 K. The inset of (**b**) represents the magnified image (**b**) near the superconducting transition.

The dependence of the electrical resistivity on temperature for the single crystal samples is illustrated in Figure 4. For Se concentration (*x*) between 0–0.12 (Figure 4a), a kink and hysteresis related to the structural phase transition to the T_d_ type can be observed during the thermal cycle at approximately 250 K at *x* = 0–0.04. This kink was suppressed gradually at *x* = 0.07 and 0.12. In addition, the temperature at which the kink was observed (*T*_s_) differed according to the substitution of Se ions. Samples demonstrating a curve kink (*x* = 0–0.04) did not exhibit a superconducting transition, as shown in Figure 4b. The rest of the samples exhibited the onset of *T*_c_ at approximately 3 K. Moreover, the resistivity drop was small (~10% from the normal state), indicating the emergence of superconductivity below 2 K. As the Se concentration increased (*x* > 0.12), the kink in the curve was entirely suppressed (Figure 4c). Furthermore, samples with *x* = 0.13–0.28 exhibited superconductivity at approximately 3 K, as depicted in Figure 4d. These results indicate that Se substitution suppressed the kink gradually and induced an increase in *T*c. Furthermore, Se substitution in polycrystalline MoTe_2_ has been reported to suppress the structural phase transition at approximately 250 K; moreover, it has been reported to result in an increase in the onset of *T*_c_ to 3 K [10]. These results are similar to the results presented herein.

**Figure 5:**
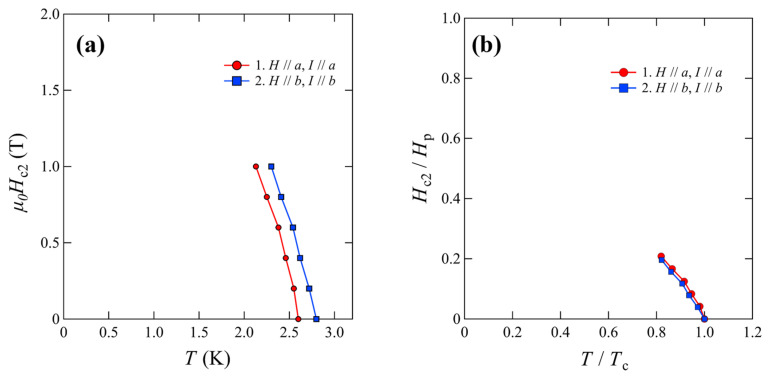
(**a**) Temperature dependence of the upper critical magnetic field. (**b**) illustrates (**a**) using normalized axes. Vertical and horizontal axes were normalized using Pauli’s limit *H*_p_ and *T*_c_, respectively.

The superconducting state of the materials was investigated using resistivity measurements under magnetic fields. The sample prepared with *x* = 0.28 was selected for this purpose. Measurements were performed by applying magnetic fields and currents along each axis because the sample exhibited different in-plane axes. Therefore, two measurements were performed when the magnetic field and current were applied along the *a*-axis and *b*-axis. Because the transition was gradually suppressed when applying a magnetic field in both measurements, it could be concluded that this transition was related to superconductivity. The temperature dependence of *µ*_0_*H*_c2_ for all measurements is summarized in Figure 5a,b. *T*_c_ at each magnetic field was defined as the temperature at which the resistivity reached 90% of the normal-state resistivity. This *T*_c_ was denoted as *T*_c_^90%^. The estimated *µ*_0_*H*_c2_ (0) values for all experimental conditions are summarized in Table 1. The value of *µ*_0_*H*_c2_^WHH^, calculated from the Werthamer–Helfand–Hohenberg (WHH) model, was 2.4–3.8 T [15,16]. The Pauli limit, denoted as *µ*_0_*H*_c_^Pauli^, was 4.8–5.4 T, which was calculated using *T*_c_^90%^. Thus, *µ*_0_*H*_c2_^WHH^ was lower than *µ*_0_*H*_c_^Pauli^ for each condition. This tendency is consistent with that observed in previously reported high-pressure and element-substitution studies [6,10,11].

**Figure 6:**
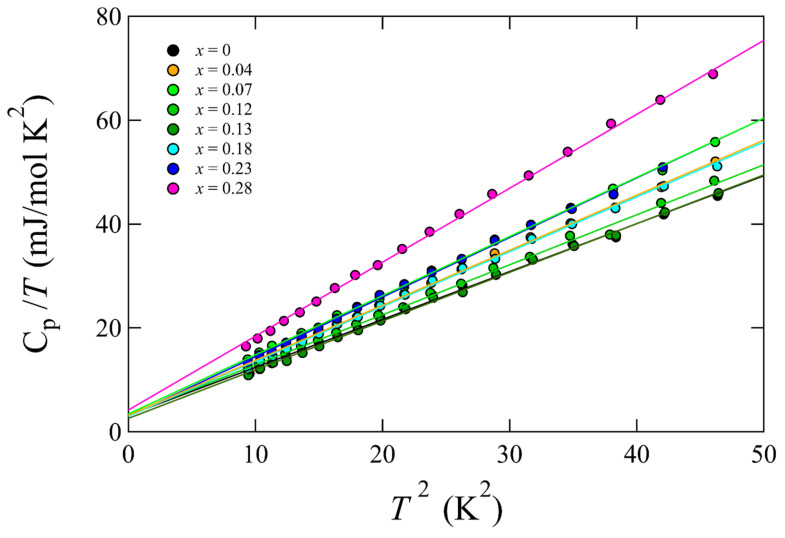
*C*_p_/*T* as a function of T^2^ for the single crystal samples. Each solid line indicates a fitting.

Specific heat measurements were performed on the Se-substituted samples to investigate structural changes and to obtain electronic structure information. The results for the low-temperature region (below 50 K) are presented in Figure 6. These data were fitted using Equation (1), which describes the temperature dependence of the metal:

*C*(*T*)/*T* = β + γ*T^2^* (1)

where β and γ denote fitting parameters. The parameters used are listed in Table 2. Neither parameter was dependent on the Se concentration except for *x* = 0.28. The samples with *x* = 0.28 exhibited slightly higher parameter values among all the other samples. However, this difference was insignificant and did not affect properties. Indeed, the metallic behavior and *T*_c_ values did not exhibit significant changes. Thus, it can be concluded that a significant change in the electronic structure does not occur due to Se substitution. This result indicates that the increase in *T*_c_ was not related to an increase in the density of states near the Fermi energy. This observation is qualitatively consistent with previously reported data for S-substituted single crystals and Se-substituted polycrystals [8,10].

**Figure 7:**
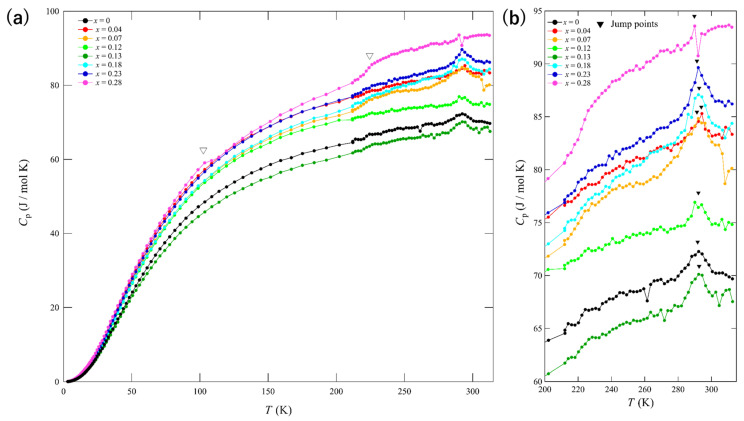
Temperature dependence of the specific heat (*C*_p_) for single crystals. (**a**) in the operating temperature range and (**b**) in the temperature range of 200–310 K. Open triangles indicate unknown small jumps. Solid triangles in (**b**) indicate *C*_p_ jumps related to structural transitions.

The temperature dependence of specific heat (*C*_p_) is illustrated in Figure 7a. It can be observed that the behavior is metallic at T < 200 K. A *C*_p_ jump can be observed at approximately 290 K until *x* = 0.23, after which it is gradually suppressed at *x* = 0.28 (Figure 7b). This temperature is consistent with the temperature corresponding to the appearance of a kink structure in the resistivity measurements for the *x* = 0–0.12 samples. Although the kink was suppressed above *x* = 0.13 in the resistivity curve, a jump in the heat capacity was observed above *x* = 0.13. These results can be attributed to either of two possibilities: phase separation in the sample or remnant of the T_d_ type. Although phase separation was not entirely excluded, composition analysis via EDX did not support this possibility. The composition of each sample (size 2–3 mm^2^) was determined based on an average of five different measurements. The measured values at each location were almost constant, which demonstrated that the substituted Se ions were uniformly distributed. It should be noted that the T_d_ type was not completely suppressed by Se substitution. Therefore, it is possible that the superconductivity observed at *x* = 0.13–0.28 appeared in the T_d_ type. The *x* = 0.28 sample exhibited a small jump at 290 K, whereas a kink and a slight jump were observed at approximately 230 K and 100 K, respectively (open triangles in Figure 7a). The sample with *x* = 0.07 also exhibited a slight kink, which indicates the appearance of new phases at this Se concentration. However, the underlying cause responsible for these phenomena requires further investigation.

**Figure 8:**
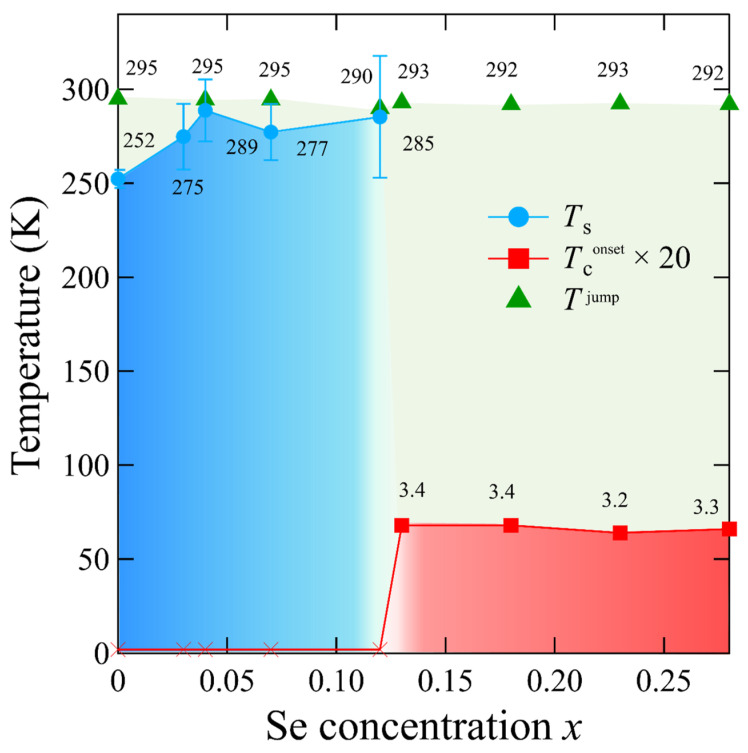
Phase diagram of the kink and superconductivity of the MoTe_2-*x*_Se*_x_* single crystal. *T*_s_ denotes the temperature that corresponds to the kink in the resistivity curve. The bars represent the temperature corresponding to the kink, which is estimated in the heating and cooling processes. *T* ^jump^ denotes the temperature attributed to the jump in specific heat. Red cross marks (×) indicate no superconductivity until 2 K.

The obtained results are summarized as a phase diagram in Figure 8 and in Table 3. Se substitution induced suppression of the kink observed in the resistivity for Se concentrations *x* > 0.12. After suppression, superconductivity at a higher *T*_c_ (approximately 3 K) occurred immediately. This result indicates that kink suppression is related to superconductivity enhancement. However, a heat capacity jump emerged, which was observed near the kink temperature at *x* = 0–0.12. This result demonstrated that phase transition to T_d_ type was not entirely suppressed, and a higher *T*_c_ phase was observed in the T_d_ type. Scanning tunneling microscopy measurements have recently been performed on Se-substituted MoTe_2_ single crystals [17]. Consequently, superconductivity was observed at *T*_c_^onset^ ~ 3 K; however, a kink structure was not observed. In contrast, a topographic image of the T_d_ type was observed at a cleaved surface. Raman measurements indicated the existence of the T_d_ type at low temperatures. From these results, it can be concluded that the observed jump in the specific heat value is presumably related to the T_d_ type transition in the bulk. Further investigations are, however, required for Se-substituted MoTe_2_ with regard to the observation of the crystal structure at low temperatures via XRD measurements or through electronic structures.

## 4. Conclusions

In this study, Se-substituted MoTe_2_ single crystals were successfully prepared and evaluated. Resistivity measurements confirmed that Se substitution suppressed the appearance of the kink and induced a relatively higher *T*_c_ (approximately 3 K). The results were analogous to those previously reported for polycrystalline structures. However, a jump in the specific heat capacity was observed at approximately 250–290 K, which was independent of the Se concentration in the crystal. In resistivity measurements, the temperature was similar to that resulting in a kink for the MoTe_2-*x*_Se*_x_* sample (*x* = 0–0.12). This result demonstrated that the T_d_ type without spatial inversion symmetry along the *c*-axis was not completely suppressed, and a high *T*_c_ was obtained in the T_d_ type. We believe that this study may be significant for investigations on the topological physics of Weyl semimetals.

## Figures and Tables

**Table 1:** *T*_c_ and *H*_c2_ values estimated by the WHH model and the Pauli limit.

*T*_c_^90%^ (K)	-d*H*_c2_/d*T*	*µ*_0_*H*_c2_^WHH^ (T)	*µ*_0_*H*_c2_^Pauli^ (T)
2.9	1.2	2.4	5.3
2.6	2.1	3.7	4.8
3.0	1.2	2.4	5.4
2.8	2.0	3.8	5.1

**Table 2:** Fitting parameters used in Figure 6.

Se Concentration (%)	β (mJ/mol K^4^)		γ (mJ/mol K^2^)	
0	3.2	(3)	0.92	(1)
0.04	3.2	(2)	1.06	(1)
0.07	3.4	(2)	1.14	(1)
0.12	3.3	(4)	0.97	(1)
0.13	2.6	(2)	0.94	(1)
0.18	3.0	(3)	1.06	(1)
0.23	3.0	(3)	1.15	(1)
0.28	4.2	(4)	1.42	(1)

**Table 3:** *T*_c_^onset^, *T*_c_^jump^, and *T*_s_ values used in Figure 8. Hyphen (-) indicates that no transition was observed. N.D. implies that no measurement was performed.

Se Concentration (%)	*T*_c_^onset^ (K)	*T*^jump^ (K)	*T*_s_ (K)		
			Cooling	Heating	Ave.
0	-	295	247	257	252
0.03	-	N. D.	257	292	275
0.04	-	295	272	305	289
0.07	-	295	262	292	277
0.12		290	253	318	285
0.13	3.4	293	-	-	-
0.18	3.4	292	-	-	-
0.23	3.2	293	-	-	-
0.28	3.3	292	-	-	-
